# Interferon-Gamma Release Assays versus Tuberculin Skin Testing for the Diagnosis of Latent Tuberculosis Infection: An Overview of the Evidence

**DOI:** 10.1155/2013/601737

**Published:** 2013-02-07

**Authors:** A. Trajman, R. E. Steffen, D. Menzies

**Affiliations:** ^1^Gama Filho University, 20740-900 Rio de Janeiro, RJ, Brazil; ^2^Montreal Chest Institute, McGill University, Montreal, QC, Canada H2X 2P4; ^3^Federal University of Rio de Janeiro, 21941-913 Rio de Janeiro, RJ, Brazil

## Abstract

A profusion of articles have been published on the accuracy and uses of interferon-gamma releasing assays. Here we review the clinical applications, advantages, and limitations of the tuberculin skin test and interferon-gamma release assays and provide an overview of the most recent systematic reviews conducted for different indications for the use of these tests. We conclude that both tests are accurate to detect latent tuberculosis, although interferon-gamma release assays have higher specificity than tuberculin skin testing in BCG-vaccinated populations, particularly if BCG is received after infancy. However, both tests perform poorly to predict risk for progression to active tuberculosis. Interferon-gamma release assays have significant limitations in serial testing because of spontaneous variability and lack of a validated definition of conversion and reversion, making it difficult for clinicians to interpret changes in category (conversions and reversions). So far, the most important clinical evidence, that is, that isoniazid preventive therapy reduces the risk for progression to disease, has been produced only in tuberculin skin test-positive individuals.

## 1. Introduction

Tuberculosis (TB) is an important cause of morbidity and mortality worldwide [1]. Governmental and non-governmental organization efforts and investments in the last decades to control the epidemic have resulted in a steady decline in disease incidence and mortality [2]. One third of the world population, however, has latent tuberculosis (TB) infection (LTBI), and to reach the United Nations Millennium Goals of eliminating the disease by 2050, it is its necessary to couple diagnosis and treatment of active disease with new approaches to reduce this vast reservoir of LTBI, sufficient for generating new TB cases for many decades even if transmission was suppressed [3]. Thus, in addition to rapid, accurate, and inexpensive detection of active TB, the detection—and treatment—of LTBI is also an important strategy for TB control [1]. In the present paper, we summarize the advantages and limitations of tuberculin skin testing (TST) and overview the evidence for the use of the newer interferon-gamma release assays (IGRA) for the diagnosis of LTBI (Table 1). 

## 2. Tuberculin Skin Testing

Until the beginning of this century, TST was the only diagnostic method for detecting LTBI. The test is based on a delayed-type hypersensitivity reaction that occurs when those infected with *M. tuberculosis* are exposed to certain antigenic components present in extracts of culture filtrates, the “tuberculins.” In this type of reaction, T cells, sensitized by prior infection, are recruited to the skin where the tuberculin was injected and release lymphokines. The result is local induration of the skin through local vasodilatation, edema, fibrin deposition, and recruitment of other inflammatory cells to the area [4]. An induration greater than 5 mm is widely accepted as a positive reaction. Different cut-off sizes can be considered. Although widely used, TST has limitations. TST sensitivity may be reduced by malnutrition, severe TB diseases and immunodeficiency, such as that related to HIV [5, 6]. Decreased TST specificity might occur in settings where nontuberculous mycobacteria (NTM) are prevalent and in populations who have received BCG vaccination after infancy, although the effect of BCG vaccination on TST reactions is very modest after 10 years or more if vaccination is given in infancy [7]. Additionally, completing the TST requires two health care visits, for tuberculin injection and induration measurement, which results in loss of reading in approximately 10% of cases [8]. Also, measurement of reaction size is subject to interobserver variability, although this is greatly reduced with adequate training [9–11]. In addition, one positive TST result does not distinguish recent from remote infection, which has a lower risk of progression to disease [12, 13]. Although TST reversions are shown to occur, they are more common in older adults, estimated at 8% per year [14]. When a tuberculin reaction reaches 10 mm or greater, the additional tests becomes uninterpretable [15], meaning that serial TST has no place in monitoring treatment response. Moreover, repeated injections of tuberculin are known to elicit the booster phenomenon [15]. For this reason, in situations where serial testing is indicated, a two-step tuberculin test is recommended at the time of first testing [16]. Finally, in low- and medium-income countries with a high-TB incidence, diagnosis of LTBI is reported to be difficult because of the extra workload brought by TST [17]. In those countries, detection and treatment of active TB is the priority, although some could invest in detection and treatment of LTBI if an easier test was available [18]. 

Yet, TST has many advantages. It is a century-old test that has been widely used, and its clinical applications are very well studied. Cut-off points have been established for indication of isoniazid preventive therapy (IPT) for different ages and risk groups [19]. More importantly, the benefit of IPT in groups with a positive TST has been widely proven, as has been the lack of benefit of IPT in TST-negative subjects [19, 20]. Finally, there are very clear definitions of conversion and boosting [15]. Conversion is defined as an induration over 10 mm with an increase of at least 6 mm over the previous result. This is because the standard deviation of repeated readings (intra- and interobserver variation and within-subject variation) is 3 mm. It is thus expected that 95% of subjects will have an increase of less than 6 mm (two standard deviations) not related to TB exposure. In summary, the main application of TST is to detect LTBI, but its role alone for evaluating the risk for progression to disease is limited. It must be interpreted in light of the clinical situation and epidemiological history to allow for evaluation of risk of disease.

## 3. Interferon-Gamma Release Assays (IGRAs)

IGRAs were designed to detect the immune response to specific *M. tuberculosis *antigens, which are not present in BCG or certain nontuberculous mycobacteria. Two tests are commercially available; one is based on the Elispot (T-SPOT.TB, Oxford Immunotec, UK) and the other on the enzyme-linked immunosorbent assays (ELISA) technique (QuantiFERON-TB Gold-in-Tube, Cellestis, Australia, QFT-GIT). Both tests are based on the principle that the T cells of an individual who have acquired TB infection will respond by secreting the cytokine interferon-gamma (IFN-*γ*) when restimulated with *M. tuberculosis* antigens [21]. To avoid cross-reactivity, these tests use antigens encoded in the region of difference 1 (RD1), a portion of the *Mycobacterium tuberculosis* genome that is absent from the genome of BCG and many NTM [21]. The QFT-GIT uses the patient's whole-blood and is based on the ELISA measurement of IFN-*γ* produced in response to three *M. tuberculosis *antigens (ESAT-6, CFP-10, and TB7.7). The second test, T-SPOT.TB, is based on the enzyme-linked immunospot measurement of the number of peripheral mononuclear cells that produce IFN-*γ* after stimulation with two mycobacterial antigens: ESAT-6 and CFP-10.

There have been several publications on IGRAs' accuracy, predictive value, cost-effectiveness, and indications since they became available commercially. Many high-income countries have incorporated these tests in their national guidelines [32]. However, the World Health Organization (WHO) has recently published recommendations against their use in low- and medium-income countries (LMIC) [33], since most IGRA studies have been done in high-income countries and mere extrapolation to low- and middle-income settings with high background TB infection rates being not appropriate. In addition, systematic reviews have suggested that IGRA performance differs in high- versus low-TB and HIV incidence settings, with relatively lower sensitivity in high-burden settings [33]. One other important aspect against their use in LMICs is the cost. Given similar performance but higher costs, IGRAs replacing TSTs as a public health intervention in settings with limited budget being not recommended.

## 4. Accuracy of IGRAs to Diagnose LTBI

The evaluation of the accuracy of these tests to diagnose LTBI has been hampered by the fact that there is no gold standard. Evaluation of new techniques, therefore, relies on the use of proxies for LTBI such as active TB, TB exposure gradients, rates of TB incidence in untreated cohorts and results of randomized clinical trials comparing the benefit of preventive treatment in patients who test positive and negative. Here, we briefly review the evidence using each of these approaches. 

### 4.1. Active TB

Most of the studies on sensitivity were performed in patients with active TB, relying on the principle that patients with active TB are necessarily infected. The main drawback of this approach is that patients with active TB usually have immunosuppression due to the disease itself or to malnutrition, hampering the ability to respond both to tuberculin and to specific *M. tuberculosis* antigens present in IGRA tests [22]. Thus, sensitivity of both TST and IGRA tests for LTBI is likely to be underestimated in these studies. Conversely, specificity has been evaluated in subjects believed not to have been exposed to TB antigens, which is difficult to prove. In a meta*­*analysis, Pai et al. [22] assessed the accuracy of IGRAs for the diagnosis of LTBI. Sensitivity was determined by using patients with newly diagnosed active TB as a surrogate of latent infection. This meta-analysis included studies performed in low- and high TB-burden countries and found a pooled sensitivity of 78% (95% confidence interval (CI), 73% to 82%) for QuantiFERON-TB Gold and 92% (CI, 90% to 93%) for T-SPOT.TB. Sensitivities of both TST and Quanti-FERON were highly heterogeneous, possibly due to HIV coinfection, advanced disease, and malnourishment, which reduce immune response. Indeed, in a study of mostly smear negative active TB patients, that is, with probably early disease, 10% were TST negative [34], while in a study in Japan, 43% of patients with advanced TB were TST negative [35]. The specificity studies were all from countries with low rate of TB incidence with varying BCG policies and found a pooled specificity of 98% (CI, 96% to 99%) for all QFT studies, with little difference among BCG and non-BCG-vaccinated populations (96% versus 99%, resp.) and 93% (CI, 86% to 100%) for T-SPOT.TB (only one study among BCG-vaccinated subjects). The TST sensitivity was similar, with a pooled estimate of 77% (CI, 71% to 82%). Specificity, however, showed a more marked difference between non-BCG and BCG-vaccinated participants. In non-BCG-vaccinated subjects, the pooled estimate was consistently high, 97% (CI, 95% to 99%) but in the BCG vaccinated population specificity was only 59% (CI, 46% to 73%). Four of the 6 studies, however, were from countries where BCG vaccination is done after infancy or done multiple times [36]. No studies have directly compared TST and IGRAs in populations that were BCG vaccinated only at birth, where specificity of TST is much higher. 

### 4.2. Exposure Gradients

Usually, TB exposure is captured either as a dichotomous variable (exposed and unexposed) or as a gradient (degree of exposure is graded according to duration, or proximity). In exposure gradient studies, contacts are assigned to 3 or more categories from most to least exposed. The proportion of positive results should parallel the exposure gradient. The best test should demonstrate the closest correlation of proportion positive to this gradient. The main challenge with these methods is the difficulty in quantifying the exact degree of exposure of contacts. In addition, sometimes the criteria for exposure are different among studies, making pooled estimates difficult. A total of 10 studies in pediatric populations used this method [37–46]. There was great variation in the definition of the exposure groups. For TST, most studies used a 10 mm cut-off point, while 2 studies used 5 mm and one study 15 mm. The overall pooled odds ratio (OR) was 1.34 (CI, 0.66–2.72), 1.93 (CI, 0.98–3.77), and 1.83 (CI, 0.67–5.02) for TST when 5 mm, 10 mm, or 15 mm were used, respectively, as the cut-off value [47]. For QuantiFERON, the pooled OR was 3.51 (CI, 1.85–6.66), and for T-SPOT it was 1.31 (CI, 0.76–2.27) [47]. All confidence intervals overlapped, indicating similar performance for all tests [47].

### 4.3. Incidence of TB in Cohorts

More important than identifying individuals with LTBI is the recognition of those who will progress to active disease, because those are the subjects who will benefit from isoniazid preventive therapy (IPT). So far, IGRAs have been shown to have a low predictive ability for progression from LTBI to active disease although individuals with positive IGRA have slightly higher risk than those with positive TST results [30, 48–51]. 

Two recent meta-analyses have reviewed the predictive value of TST and QFT [30, 31]. One review included fifteen studies comparing the incidence of active TB in IGRA-positive and TST-positive subjects [31], including studies in children, healthcare workers, and PLWHA [47, 52, 53]. Among these studies, 5 looked at the risk for progression to TB in PLWHA [54–58]. Three showed a higher risk in IGRA-positive compared to IGRA-negative subjects [54–56], but 2 did not [57, 58]. By contrast, the risk of developing active TB in TST-positive PLWHA is well documented. Moreover, the benefit of IPT in TST-positive PLWHA has been confirmed in many studies [20, 59]. Overall, the incidence rate of TB over a median time of 4 years was very low in both TST- and IGRA-positive test subjects (3.7 to 4.8 and 1.0 to 4.5 per 1000 persons-year, resp.). These low incidence rates show that both tests have a low predictive value for active disease.

The second review on the predictive value of IGRAs [30] included more studies, since it did not exclude those in which IGRA results were not blinded to the treating physician, ignoring the risk of “incorporation bias” of these studies. Although the authors found that progression rates for commercial IGRAs were significantly higher than those for the TST (2.7% versus 1.5%), they also concluded that they are both low in absolute numbers [30].

### 4.4. Evidence of Benefit from Isoniazid Preventive Therapy

More important than establishing the accuracy and predictive value of these tests is to evaluate the benefit from IPT in reducing the risk for progression to active disease in subjects with a positive result. While there is very strong evidence of the protection from IPT in TST-positive subjects [19, 20], so far there are no studies showing direct evidence of the efficacy of IPT based on IGRA results. Indirect evidence of this benefit is based on isoniazid protection against TB in patients who have a positive TST. A recent study in Botswana has shown that long-term IPT in TST-positive persons living with HIV/AIDS (PLWHA) has a strong impact on survival. The study has also shown that isoniazid has little benefit in PLWHA TST-negative individuals [20, 59]. 

TST conversion usually reflects recent infection. The risk of progression to disease in recent converters is still higher, thus, IPT is recommended, regardless of HIV status or background of TB incidence. However, for PLWHA who become TST positive after initiating highly active antiretroviral therapy (HAART), conversion should be interpreted with caution, as it may be due to immune reconstitution and not recent infection. 

Given the similarity of TST and IGRAs in cohort studies, it is reasonable to believe that IPT in IGRA-positive individuals should provide at least the same benefit as in TST-positive individuals [60], but the lack of benefit in IGRA-negative individuals is yet to be evaluated.

## 5. IGRA as a Marker of Cure Following Treatment of LTBI

As previously remarked, TST reversion is not associated with successful treatment of LTBI. To test the usefulness of IGRAs to monitor TB and LTBI treatment, IGRA tests have been applied serially during and after treatment of patients with active TB and of healthy subjects undergoing LTBI treatment [61–76]. Overall, results were highly contradictory, with increases or decreases of interferon-gamma levels being observed randomly, regardless of clinical response to treatment [77]. Chee et al. [63] compared QFT-GIT and the T-SPOT.TB at baseline, after treatment completion and after 6 months in sputum-positive, immunocompetent patients. Although there was a significant rate of reversions (13.9% for T-SPOT.TB versus 39.2% for QFT-IT), a  substantial proportion of patients remained test positive 6 months after TB treatment (79% and 46% with T-SPOT.TB and QFT-GIT, resp.). There are no long-term studies of IGRA in the followup of subjects treated for LTBI, but a few short-term studies have shown that a decrease in quantitative results can occur after IPT, regardless of reversion. Dyrhol-Riise et al. [71] tested the performance of QFT-GIT for monitoring LTBI treatment in patients before and three and 15 months after the beginning of isoniazid and rifampicin treatment. The IFN-*γ* responses were comparable at the three time points, with no statistically significant change during the course of treatment. A recent meta-analysis supports these findings [77]. In summary, IGRAs have, at present, no place in monitoring response to treatment of TB or LTBI.

## 6. LTBI in Special Populations 

The accuracy of IGRA tests have been tested in distinct subpopulations such as children [38, 47, 48, 56, 78–84], PLWHA [23, 29, 39, 40, 47, 54, 55, 85–103], other immunosuppressed patients (those with renal disease or in use of tumor necrosis factor-alpha inhibitors), [104–106] and healthcare workers [53]. 

### 6.1. Immunosuppressed Patients

As in immunocompetent populations, the sensitivity of IGRA tests in PLWHA has also been analyzed in patients with active TB. In 18 studies, 16 of them in LMIC [52], sensitivities of both TST and IGRA tests were lower in PLWHA because both require an adequate immune response. Pooled sensitivity of QFT-GIT was 60% (CI, 34 to 82%). T-SPOT.TB seemed slightly less affected by immunosuppression, with a pooled sensitivity of 76% (CI, 45% to 92%) [18], but only five studies compared their sensitivity head to head [93, 107–110], with only one study showing QFT-GIT with a greater sensitivity than T-SPOT.TB in PLWHA [110]. Likewise, 14 studies have analyzed the role of IGRA in patients with autoimmune disease [111–124], 10 of them in countries where BCG vaccination is not routinely done [111–113, 116–118, 120, 121, 123, 124]. Most studies included a small number of patients, immunosuppressive therapy was variable or not yet started, and very few were conducted in countries with high-TB prevalence. Above all, no longitudinal study to assess the risk of progression to disease was carried out in this population [106]. Finally, there are no studies on the role of IGRA tests among transplanted patients under immunosuppressive therapy.

As mentioned above, special caution is needed when interpreting conversion and reversions in PLWHA. Reversion can be a consequence of declining immunity and should alert the physician. In contrast, LTBI/TB treatment of naive TST-negative individuals with advanced HIV infection (CD4+ cell count <200/*μ*L) started on HAART should be retested for LTBI once they achieve CD4+ cell counts >200/*μ*L [125]. 

### 6.2. Children

Regarding LTBI in the youngest, although a few studies have shown a lower sensitivity of IGRA tests in very young children [39, 40, 47, 87, 126–128], the sensitivity and specificity of IGRA in older children exposed to index cases are similar than in adults, as long as they are not infected by HIV [47]. Although BCG is not expected to interfere with IGRAs, the lower sensitivity found both in TST and IGRAs among very young children might be explained by immune immaturity or by capturing a population with underlying conditions that may interfere with immunity, such as coinfections with helminths and malnutrition [129]. Overall, the sensitivity and specificity among all children with active TB were similar for TST, QFT-G/QFT-GIT (QFT), and T-SPOT.TB, with a pooled sensitivity of 80% (CI, 70% to 90%), 83% (CI, 75% to 92%), and 84% (63% to 100%), respectively. Pooled specificity for TST, QFT, and T-SPOT.TB was 85% (CI, 63% to 100%), 91% (CI, 78% to 100%), and 94% (87% to 100%), respectively [47]. 

### 6.3. Highly Exposed Populations and Serial Testing

Highly exposed groups, such as health care workers and prisoners, as well as patients at high risk for disease, such as PLWHA, are screened for LTBI every 6 to 12 months. Although it has been described that tuberculin injection may also result in boosting of IGRA responses, this effect is not seen if QFT-GIT or T-SPOT.TB is done within 3 days of performing the TST [24, 130]. 

Unlike TST, which should only be repeated if previously negative, IGRA tests may be repeated, regardless of their previous results. However, serial testing subjects not undergoing treatment indicate that there are high rates of spontaneous reversions and conversions, although it is always harder to know if conversion is spontaneous or a consequence of real TB infections [23, 131–135]. Other possible explanations for a conversion would be nonspecific infections and vaccines, laboratorial technique variability, such as blood withdrawal preparation, delay to and time of incubation, and lot of the kit. Biological variables might also interfere. Although there is some evidence that QFT-GIT conversion is related to a greater risk of progression to TB [136], the predictive value of IGRA (QFT) conversion for the development of TB disease is still controversial, and fluctuations in IFN-*γ* responses among serially tested individuals reported in longitudinal studies remain unexplained and nonspecific.

In addition, spontaneous reversions are more likely to occur in subjects with borderline results, close to the cut-off value, are not related to treatment, and are more likely if TST negative [24, 137–140]. In such cases, it has been interpreted as self-clearance of infection, but there is not enough evidence for such a conclusion [71, 125, 141]. For these reasons, serial testing with QFT-GIT and T.SPOT-TB should be considered unreliable and is not recommended at least until the conversion and reversion phenomena are better understood.

## 7. Conclusions and Recommendations

There is still a lot to be learned about IGRAs. Until then, IGRAs can be interpreted similarly to TST for the diagnosis of LTBI; with the exception of populations BCG vaccinated after infancy, IGRAs have a better positive predictive value because of their higher specificity.

More importantly, although both TST and IGRAs are useful for detecting LTBI, neither test is all that accurate for predicting risk for disease alone, and positive tests must be interpreted in light of other clinical risk factors. LTBI testing screening is recommended only for those with a known risk factor for progression to disease, such as recent infection, young age, and immune suppression. Clinicians can be confronted with discordant TST and IGRA results and be unsure of how to manage these patients. Because protection from IPT is well established only among TST+ subjects, and because spontaneous reversion of positive IGRA is very common if the TST is negative, the authors recommend not treating TST−/IGRA+ individuals, unless they have very high risk for disease, as in HIV-positive individuals, and are clearly exposed to TB, in which case treatment for LTBI should be considered [142].

Physicians still need to rely on clinical and epidemiological grounds in order to decide if IPT is indicated in an individual patient with a positive TST or IGRA. This decision should take into account the presence of risk factors for development of disease and the risk for hepatotoxicity from IPT, which increases with age. Patients' preferences should also be considered. To help physicians decide, an interesting tool has been developed and is accessible at http://www.tstin3d.com [12].

As for treatment monitoring and other uses of serial testing, more knowledge is needed before we can incorporate IGRAs in clinical practice. 

## Figures and Tables

**Table 1:** Comparison of TST and IGRA regarding several tests' characteristics.

Test characteristics	TST	QFT	T-SPOT.TB
Overall sensitivity [22]	77%	78%	92%
HIV uninfected [9]		84%	88%
HIV infected [18]		65%	68%
Overall specificity [22]	97%	98%	93%
HIV uninfected		NA	NA
HIV infected [18]		52%	61%
Decreased specificity if BCG vaccinated?	Yes, particularly if done after infancy or repeatedly [22]		No effect [22]
Prone to nonspecific variations in test results?	Yes, from reader variability [10]		Yes, from innumerous factors [23–28]
Thresholds for conversions evidence based?	Yes [15]	No	No
Prone to conversions?	Yes, mostly due to TB exposure		Yes (high when only negative-positive definitions are used) [29]
Prone to reversions?	Yes [29]		High in individuals with weak IGRA positivity and discordance with TST [29]
Boosting?	Yes [15]		Yes, possibly if given 3 days after performing TST [24]
Conversion associated with risk of progression to active TB?	Yes (strong evidence)		No direct evidence
Ability to detect those at high risk of developing active TB	Weak [30, 31]		Weak [30, 31]
Preventive treatment of individuals with conversion reduces risk of progression to active TB?	Yes [19, 20]		No evidence
